# Promoter Hypermethylation of the *EMP3* Gene in a Series of 229 Human Gliomas

**DOI:** 10.1155/2013/756302

**Published:** 2013-09-03

**Authors:** Marta Mellai, Angela Piazzi, Valentina Caldera, Laura Annovazzi, Oriana Monzeglio, Rebecca Senetta, Paola Cassoni, Davide Schiffer

**Affiliations:** ^1^Neuro-Bio-Oncology Center, Policlinico di Monza Foundation (Vercelli)/Consorzio di Neuroscienze, University of Pavia, Via Pietro Micca, 29, 13100 Vercelli, Italy; ^2^Department of Medical Sciences, University of Turin, Via Santena 7, 10126 Turin, Italy

## Abstract

The epithelial membrane protein 3 (*EMP3*) is a candidate tumor suppressor gene in the critical region 19q13.3 for several solid tumors, including tumors of the nervous systems. The aim of this study was to investigate the *EMP3* promoter hypermethylation status in a series of 229 astrocytic and oligodendroglial tumors and in 16 GBM cell lines. The analysis was performed by methylation-specific PCR and capillary electrophoresis. Furthermore, the EMP3 expression at protein level was evaluated by immunohistochemistry and Western blotting analysis. Associations of *EMP3* hypermethylation with total 1p/19q codeletion, *MGMT* promoter hypermethylation, *IDH1/IDH2* and *TP53* mutations, and *EGFR* amplification were studied, as well as its prognostic significance. The *EMP3* promoter hypermethylation has been found in 39.5% of gliomas. It prevailed in low-grade tumors, especially in gliomas with an oligodendroglial component, and in sGBMs upon pGBMs. In oligodendroglial tumors, it was strongly associated with both *IDH1/IDH2* mutations and total 1p/19q codeletion and inversely with *EGFR* gene amplification. No association was found with *MGMT* hypermethylation and *TP53* mutations. In the whole series, the *EMP3* hypermethylation status correlated with 19q13.3 loss and lack of EMP3 expression at protein level. A favorable prognostic significance on overall survival of the *EMP3* promoter hypermethylation was found in patients with oligodendroglial tumors.

## 1. Introduction 

The epithelial membrane protein 3 (*EMP3*) is a myelin-related gene that belongs to the peripheral myelin protein 22-kDa (PMP22) gene family of small hydrophobic membrane glycoproteins. It includes four closely related members (PMP22, EMP1, EMP2, and EMP3), as well as the additional and more distant member MP20 [1–3].

The human *EMP3* gene maps on chromosome 19q13.3 [4]. It encodes for a 163-amino acid protein that contains 4 transmembrane domains and 2 N-linked glycosylation sites in the first extracellular loop. The *EMP3* amino acid homology with the peripheral proteins PMP22, EMP1, EMP2, and MP20 is 41, 33, 38, and 23%, respectively. The highest homology occurs in the transmembrane domains.

Based on the suggested functions of PMP22, *EMP3* may be involved in cell proliferation, cell-cell interactions, and apoptosis. It is expressed in most tissues, especially in peripheral blood leukocytes, ovary, intestine, and various embryonic tissues [2, 3].

The *EMP3* gene has been proposed as a candidate tumor suppressor gene (*TSG*) on 19q13.3 in several human solid tumors, such as gliomas, neuroblastoma, esophageal squamous cell carcinoma (ESCC), breast cancer, and pheochromocytoma [5–12]. In these malignancies, it is frequently inactivated by a hypermethylation-mediated transcriptional gene silencing. The latter is restored by the demethylating agent 5-aza-2-deoxycytitidine in a large collection of human neuroblastoma [5].

The *EMP3* expression levels and the hypermethylation frequencies among different malignancies have been investigated [5–12]. Within tumors of the nervous system, DNA hypermethylation and aberrant expression of the *EMP3 *gene have been reported in both gliomas (24%) and neuroblastoma (39%) [5]. In the latter, the *EMP3* hypermethylation may have a clinical relevance because it is associated with poor survival at two-year follow-up and with a higher mortality rate [5]. 

In gliomas, different methylation frequencies among histological types have been reported. By methylation-specific polymerase chain reaction (MS-PCR), a hypermethylation in the CpG island of the *EMP3* promoter region has been found in 83% and 84% of WHO grades II and III astrocytomas, respectively; in 80% and 73% of WHO grades II and III oligoastrocytomas, respectively; and in 73% and 78% of WHO grades II and III oligodendroglial tumors, respectively [7]. *EMP3* is hypermethylated in 17% of primary GBMs (pGBMs) and in 89% of secondary GBMs (sGBMs), respectively [7]. These observations have been confirmed by other studies [8–17]. Normal nervous tissue showed neither *EMP3* hypermethylation nor lack of mRNA expression [10].

The aim of this study was to investigate the *EMP3 *promoter hypermethylation status, as well as the EMP3 expression at protein level, in a large series of 229 human gliomas and in 16 GBM cell lines. Associations of *EMP3* promoter hypermethylation with total 1p/19q codeletion,* MGMT* promoter hypermethylation, *IDH1/IDH2 *and *TP53 *mutations, and *EGFR* amplification were studied. The prognostic role of the *EMP3 *promoter hypermethylation was investigated. 

## 2. Materials and Methods 

### 2.1. Patients

Formalin-fixed paraffin-embedded (FFPE) brain tumor samples were collected from a total of 229 patients (Table 1). Tumors were surgically removed at the Neurosurgery Unit, Department of Neuroscience, University of Turin (Turin, Italy). The study was approved by the relevant Ethics Committees. The histological diagnosis was performed according to World Health Organization (WHO) guidelines [18]. Patients underwent either partial or total resection. Their demographic data are illustrated in Table 1. After informed consent, their tumor and blood/saliva samples were collected for both genetic analysis and research purposes. 

GBMs were considered as pGBM or sGBM according to a previous histologically verified low-grade glioma. 

A panel of 16 established cell lines from primary cultures of 14 pGBMs was included in the study.

### 2.2. Patient Stratification

Oligodendroglioma patients were stratified according to the therapeutic treatment received. Of 42 patients with WHO grade II oligodendroglioma, 10 received postoperative standard radiotherapy (RT) (60 Gy total dose in 27–30 fractions by LINAC) and chemotherapy (CHT), with Temozolomide (TMZ) (8/10), TMZ + PCV (1/10), and PCV (1/10). Three patients received RT alone. Chemotherapy with TMZ was administered to nine patients whereas only two received either the combined treatment of TMZ + PCV or Fotemustine. Four patients did not receive therapies. 

Of 31 patients with WHO grade III oligodendroglioma, 11 received both RT and CHT, with either TMZ (8/11) or TMZ + PCV (3/11). Two patients received either RT or CHT with TMZ alone whereas two patients had no treatment.

For 30 cases (14 WHO grade II and 16 WHO grade III oligodendroglioma patients) clinical information and follow-up were not available.

GBM patients were stratified as follows. Forty-three patients received postoperative standard fractionated radiotherapy (60 Gy total dose; 2 Gy × 5 days/week for 6 weeks). Twenty-three of 43 irradiated patients received concomitant chemotherapy with TMZ 75 mg/m^2^/daily for 6 weeks, followed by adjuvant TMZ 200 mg/m^2^ from day 1 to day 5 every 4 weeks for 6–12 cycles. Twenty patients received RT alone whereas only one patient received TMZ alone. Seven patients had no treatment. For 19 cases, follow-up was not available.

### 2.3. Isolation of Genomic DNA

Genomic DNA (gDNA) was extracted from FFPE tumor samples by a standard phenol-chloroform procedure. Prior to DNA extraction, for each sample only tumor areas previously identified as proliferating by haematoxylin and eosin (H&E) staining and microscopic examination were selected. gDNA from cell lines and peripheral blood/saliva was isolated by commercial available kits (Qiagen, Hamburg, Germany). 

### 2.4. EMP3 and MGMT Promoter Hypermethylation Status

**Figure 1:**
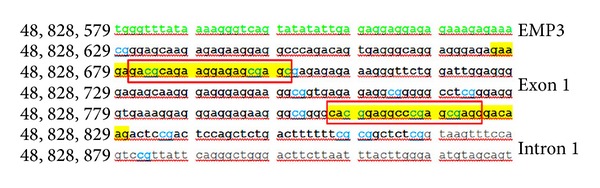
The CpG island in the promoter region of the EMP3 gene. Position of *forward *and *reverse *primers corresponding to the methylated (underlined in red) and unmethylated (underlined in yellow) sequences, respectively, in the CpG island in the promoter region of the EMP3 gene (GenBank accession number NM_001425). The 5′-UTR region is indicated in green, exonic sequences in black and intronic sequences in gray. The CpG dinucleotides are reported in blue.

**Figure 2:**
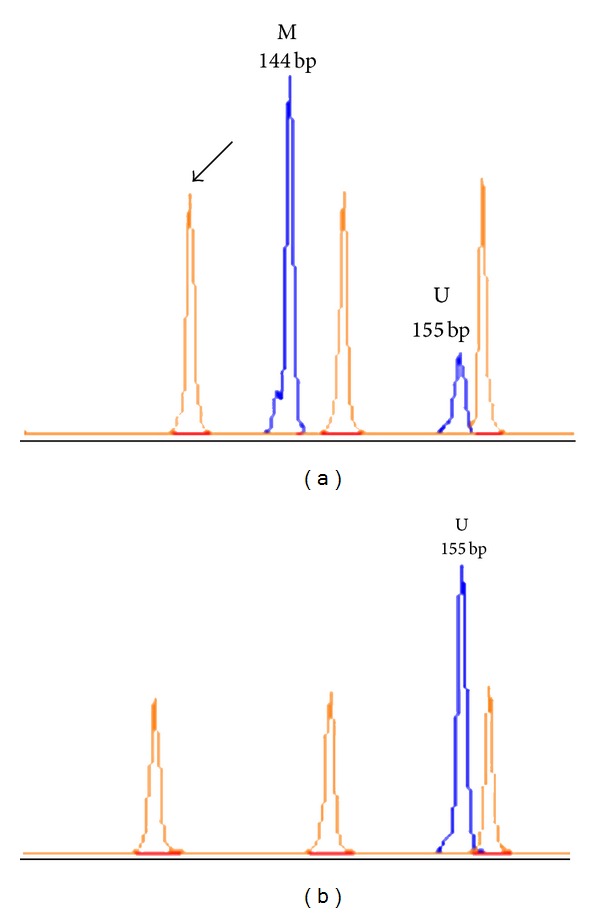
Fragment analysis of methylation-specific PCR (MSP) for the *EMP3* gene promoter. (a) Electropherogram of a tumor sample with *EMP3* promoter hypermethylation. (b) Electropherogram of a tumor sample without *EMP3* promoter hypermethylation. The 144-base pair peak refers to the methylated allele (M) and the 155-base pair peak to the unmethylated allele (U). Arrow indicates the GeneScan-500 LIZ Size Standard (Applied Biosystems).

The methylation status of genes of interest was assessed by MS-PCR followed by capillary electrophoresis (CE) [19]. One *μ*g of gDNA was subjected to sodium bisulfhite modification with the MethylEasy Exceed Rapid DNA Bisulfite Modification Kit (Human Genetic Signatures Pty Ltd, Macquarie Park, Sydney, Australia), according to the manufacturer's instruction. CpGenome Universal Methylated DNA (Chemicon International Inc., Temecula, CA, USA) and normal lymphocyte DNA were used as methylated and unmethylated controls, respectively. The primer sequences for the *MGMT* gene (GenBank accession number NM_002412) have been described previously [20]; those for the *EMP3* gene (GenBank accession number NM_001425) are depicted in Figure 1 [5]. MS-PCR was performed in a total volume of 10 *μ*L with AmpliTaq Gold 360 DNA Polymerase (Applied Biosystems, Foster City, CA, USA). After CE on an ABI 3130 Genetic Analyzer (Applied Biosystems), data were collected using GeneMapper v4.0 software for fragment analysis (Applied Biosystems). Amplicons for the *EMP3 *methylated and unmethylated allele corresponded to a 144- and 155-base pair peak, respectively. The peak height ratio between peaks for the methylated and unmethylated allele (mean of two replicates) was considered, and values >0.1 were scored as evidence of the methylated status of the *EMP3 *gene (Figure 2). 

### 2.5. EGFR Amplification Status

*EGFR* amplification status was assessed by PCR coamplification of both a 110 bp DNA fragment of the *EGFR *gene (GenBank accession number NM_005228) and a 85 bp DNA fragment of the *INF-*γ** gene (GenBank accession number NM_000619), as reference house-keeping gene. PCR conditions and fragment analysis have already been described [21]. 

### 2.6. IDH1, IDH2, and TP53 Mutation Analysis

Search for sequence variations in exon 4 of the *IDH1 *(GenBank accession number NM_005896) and *IDH2 *genes (GenBank accession number NM_002168) and in exons 4–8 of the *TP53* gene (GenBank accession number NM_000546) was performed as previously reported [22]. 

### 2.7. Direct Sequencing

All the amplicons for *IDH1*, *IDH2*, and *TP53 *genes were analyzed by direct sequencing using the BigDye Terminator v1.1 Cycle Sequencing Kit (Applied Biosystems). Data were collected by the Sequencing Analysis v.5.3.1 software (Applied Biosystems). All the identified sequence variations were confirmed with at least two independent PCR and sequencing experiments. Mutation nomenclature is in agreement with HUGO recommendations (http://www.hgvs.org/mutnomen/recs-prot.html). The reported nucleotide and amino acid numbering is relative to the transcription start site (+1) corresponding to the A of the ATG on the corresponding GenBank reference sequences. The somatic origin of each putative sequence variation was verified by the analysis of the patient constitutive DNA, when available.

### 2.8. Bioinformatic Analysis

Putative functional effects of the identified *TP53 *missense mutations were determined by *in silico* prediction using PMUT (http://mmb.pcb.ub.es/PMut/), PolyPhen (http://genetics.bwh.harvard.edu/pph/), and SNAP (https://rostlab.org/services/snap/) programs. 

The effect of missense, synonymous, and intronic variants on splicing was evaluated using NNSplice (http://biologyhelp.awardspace.com/desc7.php?id=14&type=biotech) and SpliceView (http://bioinfo2.itb.cnr.it/sun/webgene) software.

### 2.9. Chromosomal Status of 1p/19q Regions

Multiplex Ligation-dependent Probe Amplification (MLPA) was used to assess allelic losses on 1p and 19q chromosomes, as described in [21]. Analysis was performed using the SALSA-MLPA Kit P088 (lot number 0608) (MRC-Holland, The Netherlands), according to the manufacturer's instructions. Fragment analysis was performed on an ABI 3130 Genetic Analyzer (Applied Biosystems) and data were collected by the GeneMapper v4.0 software (Applied Biosystems). In each run, at least four reference samples were included for normalization. Data were analyzed using Coffalyser v9.4 software (MRC-Holland).

Threshold values to detect losses or gains in tumor samples were set at 0.75 and 1.4, respectively [21]. Ratio of adjacent probes has been considered to assess the occurrence of losses or gains. 1p and 19q chromosomes were considered to be completely deleted if all consecutive probes on 1p or 19q showed a ratio <0.75. In contrast, partial loss on 1p was defined as evidence of telomeric or interstitial deletions interesting at least two consecutive probes.

### 2.10. Immunohistochemistry (IHC)

Immunohistochemistry was performed on 5 *μ*m-thick sections with the anti-human EMP3 mouse monoclonal antibody (clone 3D4, 1 : 350, Abnova, Taipei City, Taiwan) on a Ventana Full BenchMark automatic immunostainer (Ventana Medical Systems, Tucson, AZ, USA). The UltraView Universal DAB Detection Kit was the revelation system. Heat-induced epitope retrieval (HIER) was performed in Tris-EDTA, pH 8 (Ventana). 

### 2.11. Protein Extraction and Western Blotting (WB)

Whole protein extracts from cells and FFPE tissues were isolated using a lysis buffer supplemented with a Protease Inhibitor Cocktail (Sigma Aldrich Co., St. Louis, MO, USA), 1 mM phenylmethanesulfonyl fluoride (PMSF), 2 mM sodium orthovanadate, and 10 mM sodium fluoride. Tissues were sonicated with three 10 s bursts. After protein assay (BCA Kit, Pierce Biotechnology, Rockford, IL, USA), 30 *μ*g proteins for cell analysis, and 70 *μ*g proteins for tissue analysis were separated on a 12% sodium dodecyl sulfate (SDS)-polyacrylamide gel and transblotted onto a nitrocellulose membrane. The blot was blocked in PBS containing 0.1% Tween 20 and 5% bovine serum albumin (BSA) at room temperature for 1 hour and then probed with the EMP3 monoclonal antibody (1 : 250) used for IHC, followed by treatment with horseradish peroxidase (HRP) conjugated secondary antibody (Dako, Carpinteria, CA, USA). Proteins were visualized by enhanced chemiluminescence using Immobilon Western kit (Millipore, Bedford, MA, USA). 

### 2.12. In Vitro Cultures

Surgical tumor tissue was processed as described in [23]. Culture conditions were Dulbecco's modified Eagle's medium (DMEM)/F-12 with 10 ng/mL bFGF (basic fibroblast growth factor) and 20 ng/mL EGF (epidermal growth factor) for neurospheres (NS), and DMEM with 10% fetal bovine serum (FBS) for adherent cells (AC). Both cultures were maintained in 5% O_2_/CO_2_ atmosphere. Human malignant glioma U87-MG and 010627 cell lines (kindly supplied by Dr Rossella Galli, DIBIT San Raffaele, Milan, Italy) were used as reference for both NS and AC.

### 2.13. Statistical Methods

Associations between categorical variables were evaluated using 2 × 2 contingency tables by the Chi-square (*χ* ^2^) or the two-tailed Fisher's exact test, as appropriate. 

Overall survival (OS) was defined as the time between the histological diagnosis and patient's death or last follow-up. Survival curves were estimated using the Kaplan-Meier method and differences between them were compared by the Log-Rank test (Mantel-Cox). A multivariate analysis with the Cox proportional-hazards regression model was performed for the following variables: age (≤40 or >40 years), histologic tumor grade, and the molecular variables emerged as significant by univariate analysis (*IDH1/IDH2 *mutations,* EMP3* promoter hypermethylation, total 1p/19q codeletion and *EGFR* amplification).

Analysis was carried out by SPSS v17.0 software (SPSS Inc., Chicago, IL, USA). 

## 3. Results 

### 3.1. EMP3 Methylation Status and Clinical Variables

The *EMP3 *methylation status was successfully determined by MS-PCR in 193 of 229 gliomas (84.3%). The *EMP3* promoter hypermethylation was detected in 77 of 195 tumors (39.5%). Its frequency in tumor types is reported in Table 2. It was not associated with sex, patient age (≤40 or >40 years), or tumor location. 

Among the histological types, the frequency of *EMP3* hypermethylation was higher in tumors with an oligodendroglial component, as oligoastrocytomas (14 of 20 cases, 70%) and oligodendrogliomas (46 of 73, 63%), than in astrocytic tumors (18 of 100 cases, 18%), with statistical significance (*P* < 0.0001) (Table 2).

In oligodendroglial tumors, the *EMP3 *gene was hypermethylated in 33 of 42 WHO grade II tumors (78.6%) and in 13 of 31 WHO grade III tumors (41.9%) (Table 3). In oligoastrocytomas, it was hypermethylated in nine of 12 WHO grade II tumors (75%) and in five of eight WHO grade III tumors (62.5%) (Table 3).

In astrocytic tumors, the frequency of *EMP3 *hypermethylation was as follows: 22.2% in pilocytic astrocytomas, 33.3% in WHO grade II astrotcyomas, and 37.5% in WHO grade III astrocytomas (Table 3). In GBMs, the frequency was significantly higher in secondary (three of three cases, 100%) than in primary tumors (five of 62 cases, 8.1%) (*P* < 0.0001). Both NS and AC from primary cultures of pGBMs did not show *EMP3* promoter hypermethylation.

After patient stratification for the histologic tumor grade in astrocytic and oligodendroglial tumors, the *EMP3* promoter hypermethylation was significantly more frequent in WHO grade II (45 of 63, 71.4%) than in WHO grade III tumors (21 of 47, 44.7%) (*P* = 0.0001).

Normal brain tissue and lymphocytes were completely unmethylated.

### 3.2. MGMT Methylation Status

The* MGMT *promoter hypermethylation status was assessed in 90 of 171 cases (52.6%). Its frequency in tumor types is reported in Table 4. Details are available elsewhere [21].

### 3.3. EGFR Amplification

*EGFR *amplification was identified in 23 of 61 GBMs (37.7%) and in one of the seven WHO grade III astrocytomas (14.3%). It was not found in WHO grade II astrocytomas or pilocytic astrocytomas (Table 4). 

In oligodendrogliomas, it was detected in one of 30 WHO grade II (3.3%) and in nine of 31 WHO grade III tumors (29%). In oligoastrocytomas, WHO grade II (one of four cases, 25%) but not WHO grade III tumors showed *EGFR* gene amplification.

### 3.4. IDH1 and IDH2 Mutations

Somatic point mutations at hot-spot codons Arg132 (R132) of the *IDH1* gene and Arg172 (R172) of the *IDH2* genes were identified in 61 of 178 gliomas (34.3%). Their frequency in tumor types is reported in Table 4. All mutations affected codon R132 of the *IDH1 *gene, with the exception of one oligodendroglial tumor with mutation at codon R172 of the *IDH2* gene. The spectrum of *IDH1/IDH2* mutations is available elsewhere [24].

### 3.5. TP53 Mutations

The* TP53 *mutation status was investigated in 116 gliomas and mutations were identified in 32 of them (27.6%). The *TP53* mutation rate in tumor types is reported in Table 4. The spectrum of *TP53* mutations in low- and high-grade tumors has been already described [21].

### 3.6. Chromosomal Status of 1p and 19q Regions

The 1p/19q status was assessed in 65 oligodendroglial and in 94 astrocytic tumors. The frequency of the total 1p/19q codeletion in tumor types is reported in Table 4. It was identified in 31 of 65 oligodendroglial tumors (47.7%), in two of nine oligoastrocytomas (22.2%), and, within astrocytic tumors, in only one diffuse astrocytoma. 

In oligodendrogliomas, total 1p/19q codeletion prevailed in WHO grade II tumors (18 of 32 cases, 56.3%) upon WHO grade III tumors (13 of 33 cases, 39.4%). 

### 3.7. EMP3 Hypermethylation Status and Molecular Markers

Patient stratification for the histological type revealed that *EMP3* promoter hypermethylation was significantly associated with* IDH1/IDH2* mutations in astrocytic tumors (*P* = 0.0088), GBMs included (*P* = 0.0012), in oligodendroglial tumors (*P* = 0.0006), and in oligoastrocytomas (*P* = 0.0095). 

No association was found with *MGMT* promoter hypermethylation and *TP53 *mutations either in oligodendrogliomas or in the whole tumor series. In contrast, an inverse significant correlation was identified with the *EGFR *gene amplification (*P* = 0.004) in both oligodendroglial tumors and the whole series of gliomas (*P* = 0.0005).

In the whole series of 185 glial tumors, the *EMP3 *hypermethylation was associated with loss of the 19q13.3 *locus*, as defined by loss of the two consecutive MLPA probes ZNF342 and PPP1R15A, tightly flanking the *EMP3* gene (*P* = 0.0001). Of 68 deleted cases, 43 (63.2%) were methylated, in contrast to 35 of 117 (29.9%) cases without deletion. 

In oligodendroglial tumors, a significant correlation was found with the total 1p/19q codeletion (*P* = 0.0266). Furthermore, of 43 patients with total 1p/19q codeletion, 36 (83.7%) showed *EMP3* promoter hypermethylation. Among these, 30 cases (83.3%) did not display EMP3 protein expression, as detected by IHC (*P* = 0.005). 

### 3.8. EMP3 Immunohistochemistry

**Figure 3:**
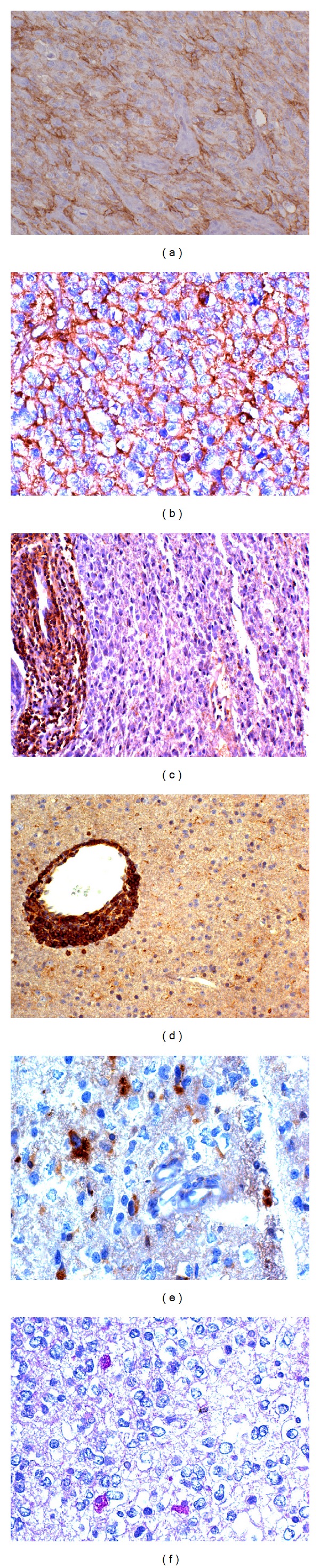
*EMP3* immunohistochemistry in astrocytic and oligodendroglial tumors. (a) GBM (unmethylated EMP3 promoter) with EMP3 cytoplasmic-cell membrane expression; DAB, 200x. (b) *id* in one WHO grade III oligodendroglioma (unmethylated EMP3 promoter); DAB, 400x. (c) WHO grade II oligoastrocytoma (methylated EMP3 promoter) with EMP3 positive perivascular infiltrates; DAB, 200x. (d) WHO grade II oligodendroglioma (methylated EMP3 promoter) with EMP3 positive perivascular cuffing of lymphocytes; DAB, 200x. (e) WHO grade II oligodendroglioma (methylated EMP3 promoter) with EMP3 positive macrophages; DAB, 400x. (f) *id* with PAS-positive macrophages, 400x.

A total of 102 WHO grade II–IV gliomas were studied. EMP3 was expressed in the cytoplasm and in the cell membrane of tumor cells, as well as in lymphocytes, macrophages, endothelial cells, and perivascular infiltrates (Figure 3). EMP3 immunoreactivity was not found in normal nervous tissue.

EMP3 immunopositivity in tumor types with respect to the *EMP3 *methylation status is reported in Table 5. In the whole series, EMP3 protein expression correlates with the latter, with statistical significance (*P* = 0.0001) (Table 5). 

### 3.9. Western Blotting

**Figure 4:**
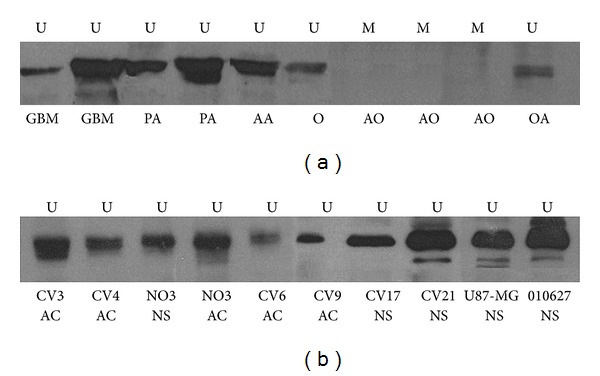
Western blotting analysis. EMP3 protein expression in (a) FFPE tumor samples and (b) GBM cell lines. U unmethylated EMP3 promoter, M methylated EMP3 promoter, PA pilocytic astrocytoma, AA anaplastic astrocytoma, GBM glioblastoma multiforme, O oligodendroglioma, AO anaplastic oligodendroglioma, and OA oligoastrocytoma.

A total of 63 FFPE tumor samples and 16 GBM cell lines (both NS and AC) were analyzed by Western blotting. Samples were scored as positive for EMP3 protein expression on the basis of a clearly visible band at 20 kDa (Figure 4). A positive correlation between EMP3 protein expression as detected by either IHC or WB analysis was found on 43 tumor samples, with statistical significance (*P* = 0.0038). 

### 3.10. EMP3 Hypermethylation Status and Survival

The correlation of *EMP3* promoter hypermethylation on patient survival was evaluated in 64 oligodendroglial tumors (38 WHO grade II and 26 WHO grade III) and in 60 GBMs.

**Figure 5:**
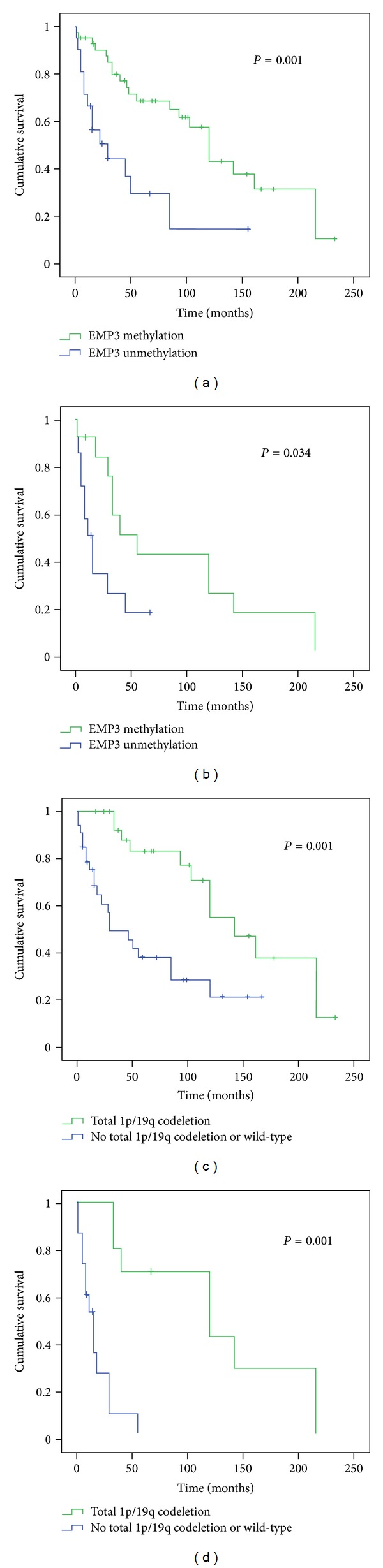
Univariate analysis for EMP3 promoter hypermethylation and total 1p/19q codeletion in oligodendroglial tumors. Kaplan-Meier survival analysis for (a) EMP3 hypermethylation versus patient overall survival (OS) in WHO grade II-III tumors, (b) EMP3 hypermethylation versus OS in WHO grade III tumors, (c), total 1p/19q codeletion versus OS in WHO grade II-III tumors, (d) total 1p/19q codeletion versus OS in WHO grade III tumors. Censored cases between EMP3 unmethylated and methylated patients are 7 and 21, respectively. Censored cases between patients with total 1p/19q codeletion and patients without total 1p/19q codeletion are 16 and 12, respectively.

In oligodendrogliomas, univariate analysis by the Kaplan-Meier method revealed that *EMP3 *promoter hypermethylation as detected by MS-PCR correlates with a significantly longer OS (*P* = 0.001) (Figure 5(a)), also in WHO grade III tumors (*P* = 0.034) (Figure 5(b)). No predictive effect of the *EMP3 *promoter hypermethylation on response to therapies was found.

Total 1p/19q codeletion was strongly prognostic on OS (*P* = 0.001) (Figure 5(c)), especially in WHO grade III tumors (*P* = 0.001) (Figure 5(d)).

Furthermore, a trend toward a positive correlation with OS was also found in GBM patients. However, the majority of GBMs was primary tumors with only five methylated patients; four censored cases were present among unmethylated patients. 

Multivariate analysis by Cox's proportional hazard regression model in oligodendroglial tumors identified total 1p/19 codeletion as the main independent prognostic factor (*P* < 0.0001), followed by the histologic tumor grade (*P* = 0.001) and the *EMP3* promoter hypermethylation (*P* = 0.071).

## 4. Discussion

In the present study the *EMP3 *promoter hypermethylation has been found in 39.5% of gliomas. It is more frequent in WHO grade II tumors (71.4%) than in WHO grade III tumors (44.7%), in agreement with previous observations [7, 16]. *EMP3* promoter hypermethylation is prevalent in oligodendroglial tumors (63%) and in oligoastrocytomas (70%) upon astrocytic tumors (18%), as already reported [5, 7, 8, 13, 14, 16, 17]. Among GBMs, its prevalence in secondary tumors is confirmed [7, 16]. Established cell lines (both NS and AC) from pGBMs do not show methylation of the *EMP3 *gene, as previously observed [15]. In pilocytic astrocytomas, *EMP3* promoter hypermethylation is rather rare.

No correlation has been found between *EMP3 *promoter hypermethylation and clinical features (sex, patient age, or tumor location), as reported [7, 13]. In contrast, the frequency of the *EMP3* promoter hypermethylation is inversely correlated with the histologic tumor grade, with statistical significance. 

The *EMP3* hypermethylation is demonstrated by the immunonegative staining of tumor cells. Wild-type tumor cells, lymphocytes, macrophages, and endothelial cells show a granular staining in the cytoplasm-cell membrane. IHC results correlate with both *EMP3* methylation status as detected by MS-PCR and WB, but in some cases there are discrepancies. Rarely, IHC is positive when *EMP3* hypermethylation is revealed by MS-PCR. This can be due to fixation defects or other unpredictable tissue events and it applies as well to the rare immunonegative cases but positive by WB analysis. Immunopositive cases with an ascertained *EMP3 *hypermethylation by MS-PCR cannot be easily explained. However, these cases are very few and do not influence the statistical correlation. The abnormality more probably concerns the tissue response than the sensitivity of the molecular assay. 

*EMP3* promoter hypermethylation is significantly associated with* IDH1/IDH2* mutations in both astrocytic and oligodendroglial tumors. The *EMP3 *gene has been proposed to belong to the CpG island methylator phenotype, recently described in gliomas (G-CIMP) [25, 26]. This phenotype, characterized by aberrant promoter methylation at multiple genes, identifies a distinct molecular subclass of glial tumors. It would prevail among low-grade tumors and it would be strongly associated with *IDH1/IDH2* somatic mutations. Furthermore, it correlates with both improved patient survival and younger age, and it is associated with the GBM Proneuronal subtype [25, 27, 28]. As a matter of fact, the G-CIMP is triggered by *IDH1/IDH2* mutations alone by remodelling both the methylome and the transcriptome [29]. In WHO grade III oligodendroglial tumors, the hypermethylated phenotype has been recently described by the EORTC study 26951 as a better predictor of survival in comparison with *MGMT* methylation [30]. 

The lack of correlation between *EMP3* and *MGMT* promoter hypermethylation found in the present series may represent the occurrence of two independent epigenetic phenomena. This is in contrast with a previous observation by unsupervised clustering analysis of the DNA hypermethylation profiles in 154 primary gliomas; of the three identified methylation patterns, Class 1 contains both the *MGMT *and *EMP3 *genes. Interestingly, Class 1 is highly methylated in 82% of low-grade astrocytic and oligodendroglial tumors, in 73% of sGBMs in contrast with 12% of pGBMs and it is significantly associated with patient OS [6].

The lack of correlation with *TP53* mutations is in agreement with the prevalence of *EMP3 *promoter hypermethylation in tumors with an oligodendroglial component upon astrocytic tumors. In contrast, the inverse association with *EGFR *amplification may be explained by its prevalence in WHO grade II tumors. 

The higher frequency of *EMP3 *promoter hypermethylation in low- than high-grade gliomas, as well as its association with other well-known early genetic aberrations, indicates *EMP3 *methylation as an early epigenetic event during gliomagenesis, preceding the differentiation of precursors. The global methylation pattern in glioma patients remains stable upon tumor progression and recurrence, as previously reported for other genes, for instance *MGMT *[25, 28, 31].

Previous clinical observations in favor of *EMP3 *as a *TSG* in gliomas were based on *EMP3* gene expression and knockdown studies, as well as on the demonstration of *EMP3 *hypermethylation as marker of poor outcome in neuroblastoma patients [5, 10]. The 19q13.3 *locus *is a critical region in both human malignant gliomas and neuroblastoma, that is frequently deleted, and associated with a specific clinical behaviour and survival rate for both tumor types [32, 33]. By cDNA microarray expression profiling, the* EMP3* promoter hypermethylation has been found to be differentially expressed in low-grade gliomas with and without 19q13.3 loss [6]. In previous studies, aberrant methylation in the promoter region of the *EMP3* gene has been found to be associated with loss of heterozygosity (LOH) on 19q13.3 in both oligodendrogliomas and neuroblastomas [5, 7]. This is confirmed in the present tumor series, as well as the significant association with total 1p/19q codeletion in oligodendroglial tumors. 

In the literature, the *EMP3* hypermethylation has been found to be significantly associated with lower transcript levels in both astrocytic and oligodendroglial tumors [7], with one exception for the latter [8], suggesting in these tumors the existence of alternative *EMP3 *epigenetic mechanisms. Furthermore, an inverse correlation between *EMP3* promoter hypermethylation and mRNA expression levels has also been demonstrated in neuroblastoma, ESCC, and breast cancer cell lines [5, 11, 12]. In the present study, the correlation between *EMP3* methylation status and transcript levels was not investigated. However, 83.3% of oligodendroglial tumors with both total 1p/19q codeletion and *EMP3* promoter hypermethylation did not display EMP3 protein expression, as detected by IHC or WB. This would be in line with the hypothesis of *EMP3* as *TSG*, because concomitant cytogenetic and epigenetic functional loss of both *EMP3 *alleles is associated with the lack of EMP3 protein expression. 

However, it must be stressed that the* CIC* gene on chromosome 19q has been recently found to be mutated in the majority of 1p/19q codeleted oligodendrogliomas, suggesting that it may be a potential *TSG* in this region [34–36]. This reduces the importance of the* EMP3* as *TSG* that, apparently, it could be just one of the several G-CIMP genes regulated by the 2-hydroxyglutarate (2-HG) increase as a consequence of *IDH1/IDH2* mutations.

The unexpected association of the *EMP3* hypermethylation with longer OS in the 64 patients with oligodendroglial tumors by univariate analysis is in agreement with a previous observation [7]. Multivariate analysis using Cox's proportional hazards regression model identifies total 1p/19q codeletion as an independent predictor of better prognosis. Therefore, the relationship between the *EMP3 *hypermethylation and the favorable prognosis may not be due to the biological consequence of the *EMP3 *gene inactivation but more probably to the prevalence in oligodendrogliomas of both total 1p/19q codeletion and *IDH1/IDH2* mutations and to their prognostic significance in these tumors [24, 37, 38]. In line with these observations, by microarray gene expression analysis, recent studies identified *EMP3 *as a new candidate gene within the 9-gene signature that is significantly associated with survival in GBM patients and they confirmed it as an independent predictor of outcome [14, 15, 39].

## 5. Conclusions 

Our observations support *EMP3 *promoter hypermethylation as an early epigenetic event in gliomagenesis, in both astrocytic and oligodendroglial tumors. It prevails in low-grade tumors, especially in gliomas with an oligodendroglial component, and in sGBMs upon pGBMs.

In the whole series, the *EMP3* hypermethylation status correlates with 19q13.3 loss and with lack of EMP3 expression at the protein level. 

In oligodendroglial tumors, it is strongly associated with both *IDH1/IDH2* mutations and total 1p/19q codeletion and inversely with *EGFR* gene amplification. No association was found with *MGMT *hypermethylation and *TP53* mutations.

The *EMP3* promoter hypermethylation correlates with statistical significance with better OS in patients with oligodendroglial tumors. This study emphasizes its relevance as a prognostic marker in gliomas.

## Figures and Tables

**Table 1:** Patient demographics.

Tumor type	WHO grading	Patients (*n*)	Gender (M/F)	Mean age (years) and range
Pilocytic astrocytoma	I	23	14/9	36 (9–68)
Diffuse and gemistocytic astrocytoma	II	14	6/8	41 (13–68)
Anaplastic astrocytoma	III	9	6/3	55 (38–78)
Primary GBM	IV	68	45/23	63 (27–81)
Secondary GBM	IV	3	3/0	45 (42–50)
Oligoastrocytoma	II	12	9/3	42 (31–63)
Anaplastic oligoastrocytoma	III	8	4/4	52 (37–71)
Oligodendroglioma	II	54	29/25	47 (26–79)
Anaplastic oligodendroglioma	III	38	23/15	55 (31–80)

**Table 2:** Frequency of EMP3 promoter methylation in glioma types.

Tumor type	Patients (*n*)	EMP3 hypermethylation (%)	*P* value
Astrocytic tumors	100	18 (18%)	
(WHO grades I–IV)			
Oligodendroglial tumors	73	46 (63%)	<0.0001
(WHO grades II-III)			
Oligoastrocytomas	20	14 (70%)	0.0001
(WHO grades II-III)			

**Table 3:** Frequency of EMP3 promoter methylation according to WHO grading.

Tumor type	Patients (*n*)	EMP3 hypermethylation (%)
*WHO grade I *		
Pilocytic astrocytoma	18	4 (22.2%)
*WHO grade II *		
Diffuse and gemistocytic astrocytoma	9	3 (33.3%)
Oligodendroglioma	42	33 (78.6%)
Oligoastrocytoma	12	9 (75%)
Total	**63**	**45 (71.4%)**
*WHO grade III *		
Anaplastic astrocytoma	8	3 (37.5%)
Anaplastic oligodendroglioma	31	13 (41.9%)
Anaplastic oligoastrocytoma	8	5 (62.5%)
Total	**47**	**21 (44.7%)**
*WHO grade IV *		
pGBM	62	5 (8.1%)
sGBM	3	3 (100%)
Total	**65**	**8 (12.3%)**

**Table 4:** Frequency of the molecular markers investigated with respect to the EMP3 methylation status.

Tumor type	EMP3 methylation status (*n*)	*IDH1*/*IDH2* mutations	*MGMT* methylation	*EGFR* amplification	Total 1p/19 codeletion	*TP53* mutations
		*n *(%)	*n* (%)	*n* (%)	*n* (%)	*n* (%)
PA	Methylated (4)	0/4 (0)	0/4 (0)	1/3 (33.3)	0/4 (0)	0/4 (0)
	Unmethylated (14)	1/14 (7.1)	4/14 (28.6)	1/10 (10)	0/12 (0)	3/14 (21.4)
DA and GA	Methylated (3)	3/3 (100)	1/2 (50)	0/3 (0)	0/2 (0)	2/3 (66.7)
	Unmethylated (6)	1/6 (16.7)	2/6 (33.3)	0/6 (0)	1/6 (16.7)	2/5 (40)
AA	Methylated (3)	3/3 (100)	1/1 (100)	0/3 (0)	0/3 (0)	2/2 (100)
	Unmethylated (5)	1/5 (20)	1/4 (25)	1/4 (25)	0/2 (0)	0/3 (0)
pGBM	Methylated (5)	0/5 (0)	2/5 (40)	2/4 (50)	0/5 (0)	1/4 (25)
	Unmethylated (57)	0/53 (0)	24/57 (42.1)	21/54 (38.9)	0/57 (0)	12/38 (31.6)
sGBM	Methylated (3)	3/3 (100)	3/3 (100)	0/3 (0)	0/3 (0)	2/3 (66.7)
	Unmethylated (0)	— (—)	— (—)	— (—)	— (—)	— (—)
OA	Methylated (9)	9/9 (100)	4/6 (66.7)	0/6 (0)	0/8 (0)	4/6 (66.7)
	Unmethylated (3)	1/3 (33.3)	1/1 (100)	0/1 (0)	0/2 (0)	1/1 (100)
AOA	Methylated (5)	3/5 (60)	4/5 (80)	1/5 (20)	1/5 (20)	1/1 (100)
	Unmethylated (3)	1/1 (100)	2/3 (66.7)	1/1 (100)	0/3 (0)	1/1 (100)
O	Methylated (33)	29/33 (77.8)	18/25 (13.3)	1/29 (3.4)	18/33 (54.5)	5/19 (26.3)
	Unmethylated (9)	5/9 (55.5)	5/9 (55.9)	1/9 (11.1)	4/9 (44.4)	1/5 (20)
AO	Methylated (13)	8/13 (61.5)	9/13 (69.2)	1/13 (7.7)	8/13 (61.5)	4/16 (40)
	Unmethylated (18)	5/16 (31.2)	12/16 (75)	8/16 (50)	3/16 (18.8)	2/13 (15.4)

PA: pilocytic astrocytoma, DA: diffuse astrocytoma, GA: gemistocytic astrocytoma, AA: anaplastic astrocytoma, GBM: glioblastoma multiforme, O: oligodendroglioma, AO: anaplastic oligodendroglioma, OA: oligoastrocytoma, AOA: anaplastic oligoastrocytoma.

**Table 5:** Correlation between the methylation status of the EMP3 gene by MS-PCR and EMP3 protein expression by IHC.

Tumor type	Total cases (*n*)	EMP3 methylation status	IHC protein expression	
			Positive	Negative
Astrocytic tumors	26	Methylated	1	7
		Unmethylated	15	3
Oligodendroglial tumors	57	Methylated	5	33
		Unmethylated	13	6
Oligoastrocytomas	10	Methylated	3	5
		Unmethylated	2	0
GBMs	9	Methylated	0	2
		Unmethylated	7	0
